# Liquid Biopsy-Based Exo-oncomiRNAs Can Predict Prostate Cancer Aggressiveness

**DOI:** 10.3390/cancers13020250

**Published:** 2021-01-11

**Authors:** Xavier Ruiz-Plazas, Antonio Altuna-Coy, Marta Alves-Santiago, José Vila-Barja, Joan Francesc García-Fontgivell, Salomé Martínez-González, José Segarra-Tomás, Matilde R. Chacón

**Affiliations:** 1 Disease Biomarkers and Molecular Mechanisms Group, IISPV, Joan XXIII University Hospital, Universitat Rovira i Virgili, 43007 Tarragona, Spain; xarupl@gmail.com (X.R.-P.); antonio.altuna@iispv.cat (A.A.-C.); martalves@hotmail.es (M.A.-S.); fontgi@yahoo.es (J.F.G.-F.); 2 Urology Unit, Joan XXIII University Hospital, 43007 Tarragona, Spain; jose.vila@urv.cat; 3 Pathology Unit, Joan XXIII University Hospital, 43007 Tarragona, Spain; mgonzalez.hj23.ics@gencat.cat

**Keywords:** exosomes, prostate cancer, exo-oncomiRNAS, TWEAK, semen

## Abstract

**Simple Summary:**

The main problem encountered in the management of prostate cancer (PCa) is the inability to distinguish slow-growing indolent tumors from aggressive tumors. It is therefore important to explore non-invasive assays for the early detection of this aggressive subtype, when it can still be treated effectively. The presence of the TWEAK cytokine in biofluids of the PCa microenvironment might drive the secretion of extracellular vesicles (EVs) containing exo-oncomicroRNAs capable of modifying the tumor microenvironment. These exo-oncomicroRNAs are potentially useful as PCa biomarkers. We identified 2 exo-oncomiRNAs isolated from semen EVs by the action of TWEAK in the tumor microenvironment and, we determined their usefulness as biomarkers of PCa prognostic. We also established, for the first time, that TWEAK modulates potential exo-oncomiRNA targets, both tightly linked to cancer progression. In conclusion, our study shows that semen detection of TWEAK-regulated exo-oncomiRNAs can improve PCa prognosis, opening new avenues for diagnosis and treatment.

**Abstract:**

Liquid biopsy-based biomarkers, including microRNAs packaged within extracellular vesicles, are promising tools for patient management. The cytokine tumor necrosis factor-like weak inducer of apoptosis (TWEAK) is related to PCa progression and is found in the semen of patients with PCa. TWEAK can induce the transfer of exo-oncomiRNAs from tumor cells to body fluids, and this process might have utility in non-invasive PCa prognosis. We investigated TWEAK-regulated exo-microRNAs in semen and in post-digital rectal examination urine from patients with different degrees of PCa aggressiveness. We first identified 14 exo-oncomiRNAs regulated by TWEAK in PCa cells in vitro, and subsequently validated those using liquid biopsies from 97 patients with PCa. Exo-oncomiR-221-3p, -222-3p and -31-5p were significantly higher in the semen of high-risk patients than in low-risk peers, whereas exo-oncomiR-193-3p and -423-5p were significantly lower in paired samples of post-digital rectal examination urine. A panel of semen biomarkers comprising exo-oncomiR-221-3p, -222-3p and TWEAK was designed that could correctly classify 87.5% of patients with aggressive PCa, with 85.7% specificity and 76.9% sensitivity with an area under the curve of 0.857. We additionally found that TWEAK modulated two exo-oncomiR-221-3p targets, *TCF12* and *NLK*. Overall, we show that liquid biopsy detection of TWEAK-regulated exo-oncomiRNAs can improve PCa prognosis prediction.

## 1. Introduction

Prostate cancer (PCa) is the most commonly diagnosed cancer and the fifth leading cause of cancer-related death in men in the developed world [1]. The incidence and morbidity of PCa continues to increase, likely due to changes in eating habits and the aging of the population [2]. A major challenge in the management of PCa is the inability to distinguish slow-growing and indolent tumors from aggressive tumors, which can lead to under-treatment of patients with aggressive tumors and over-treatment of those with indolent tumors. The prostate-specific antigen (PSA) test together with the tumor-nodes-metastasis (TNM) stage and the Gleason score of prostate biopsy [3] are considered indisputable prognostic factors to guide treatment decision-making. Among them, only PSA is objective, making it the most extensively studied biomarker in PCa [4]. However, its lack of specificity for clinically significant tumors has led to a rise in the number of prostate biopsies performed, with a consequent increase in the diagnosis of insignificant tumors and over-treatment of patients. Accordingly, the establishment of predictive biomarkers that can distinguish between aggressive and indolent PCa would be highly valuable in clinical practice, and could reduce the risk of over-diagnosis/over-treatment. In the context of biomarker discovery, liquid biopsy has proved to be a promising non-invasive modality for cancer diagnosis and prognosis that enables the assessment of circulating molecules in biological fluids, including serum, urine and semen [5].

Inflammation predisposes to the development of cancer and promotes all stages of tumorigenesis [6]. Inflammatory molecules—including cytokines and growth factors—released by immune cells of the inflammatory tumor microenvironment can have a direct effect on pre-malignant and cancer cells by enhancing their proliferation and resistance to cell death and environmental stress, thereby directly promoting tumor growth and progression [6]. Tumor necrosis factor-like weak inducer of apoptosis (TWEAK) is an inflammatory cytokine that governs tumor growth by promoting inflammation and inducing angiogenesis [7], and is produced by several cells of the immune system (natural killer cells and macrophages, among others) [8]. TWEAK can typically be found as a membrane-anchored (mTWEAK) protein on the surface of cells, but it can also be released as a soluble form (sTWEAK) by proteolytic processing. Both forms function through binding to their bona fide receptor Fn14 [7], forming a receptor–ligand pair. The role of the TWEAK/Fn14 axis has been established in some solid cancers, including breast and brain cancer [7]. We have demonstrated that low serum levels of sTWEAK in head and neck cancer are related to low survival rates, a finding that we later confirmed in a large cohort of patients, overall pointing to sTWEAK as a robust non-invasive biomarker of this disease [9,10]. We have also established a non-invasive biomarker panel with high negative predictive value to classify PCa aggressiveness that included sTWEAK levels and Fn14 mRNA expression [11].

The release of extracellular vesicles (EVs) from cells is an active process and has been shown to be a mechanism of cell-to-cell communication [12]. Exosomes are small (nanometer-size) extracellular cargo vesicles that are secreted after the fusion of endosomes with a plasma membrane, and are released by all cell types including cancer cells [13,14]. Exosomes can induce functional changes to receiving cells in the premetastatic niche—a specialized tumor microenvironment—for instance, aiding PCa cells to overcome the low-androgen conditions in distant metastatic organs [15]. Exosome secretion has long been linked to inflammation [16] and several experimental models have been employed to characterize the role of EVs in the development and progression of inflammatory diseases. The presence of sTWEAK in PCa tumors can not only contribute to fuel tumor progression [17,18], but might also promote the secretion of EVs, which will likely have an impact on the premetastatic niche, favoring the process of migration and proliferation. This is the case for exosomes derived from TWEAK-stimulated macrophages in epithelial ovarian cancer, which have been demonstrated to be internalized by the cancer cells and inhibit cell metastasis [19].

Oncogenic shuttle miRNAs (exo-oncomiRNAs), which show long-term stability in circulation and other body fluids, have been identified in exosomes [20]. Liquid biopsy exo-oncomiRNAs are thus potentially informative diagnostic and/or prognostic biomarkers and might also be helpful in understanding how tumor cells transfer their oncogenic potential to the environment [21]. Several studies have demonstrated that exomiRNAs isolated from liquid biopsy might be useful for the diagnostic and risk classification of PCa [22,23]. In this context, the most consistently reported deregulated exomiRNAs identified as promising PCa diagnostic biomarkers in both urine and blood are miR-141, miR-375, miR-21 and Let-7 [24,25,26,27]. While miR-141 is also frequently identified to be useful for risk classification in serum [27], other exomiRNAs have been proposed as having prognostic potential such as the combination of miR-1290 and miR-375 in plasma [24] and miR-2909 in urine [28]. The literature is more scarce surrounding semen, and only miRNA-342-3p and miRNA-374b-5p have been proposed as candidates for prognosis, and miRNA-142-3p and miRNA-142-5p were described as having diagnostic potential [29]. Overall, more extensive cohort studies are needed (especially using semen) to validate the identified exomiRNAs.

Exo-oncomiRNAs can be useful tools for non-invasive diagnosis and therapy monitoring in cancer; therefore, in the present study we sought to investigate whether exo-oncomiRNAs are shuttled into biofluids by the action of sTWEAK in the tumor microenvironment, and to determine their usefulness as prognostic PCa biomarkers in two different liquid biopsies: semen and post-digital rectal examination urine. We also aimed to examine the downstream targets of exo-oncomiRNAs, which might be important for the control of PCa.

## 2. Results

### 2.1. Extracellular Vesicle-Derived Exo-oncomiRNAs Are Differentially Expressed in Liquid Biopsies from Patients with Prostate Cancer Based on the Degree of Cancer Aggressiveness

We sought to search for a useful and practicable biomarker panel capable of differentiating aggressive from non-aggressive forms of PCa in liquid biopsy-based exo-oncomiRNAs isolated from EVs and secreted under sTWEAK stimulating conditions. The search was divided into two phases: the initial phase was established to isolate the EV-cargo (exo-oncomiRNAs) secreted into cell culture medium of two PCa cell lines—PC-3 and LNCaP—treated or not with sTWEAK; in the second phase, we assayed for expression using a real-time PCR array of 752 miRNA target onco-miRNAs. We specifically chose an androgen-independent line (PC-3) and an androgen-dependent line (LNCaP). Although the two cell lines do not cover the entire spectrum of PCa, they allowed us to implement a first approach to identify possible exo-miRNAs expressed through the influence of TWEAK [30].

**Figure 1:**
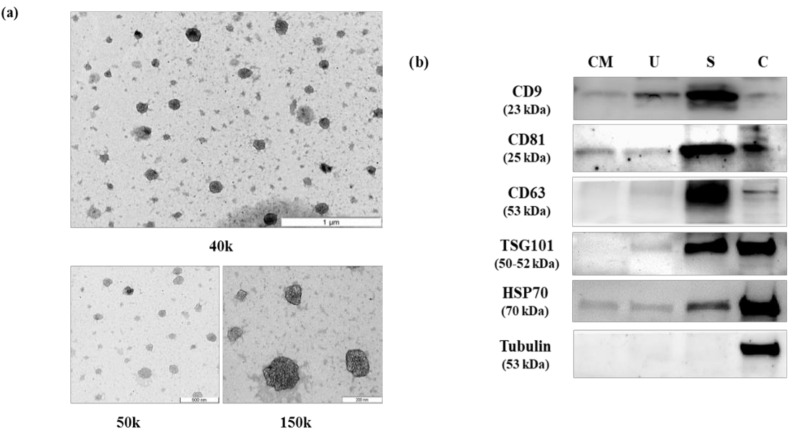
Characterization of isolated extracellular vesicles. (**a**) Analysis of extracellular vesicles (EVs) by electron microscopy at different magnification. (**b**) Western blot image of protein extracts prepared from EVs isolated from PC-3 culture media (CM), post-digital rectal examination urine (U), semen (S) and total cell extract from PC-3 (C), and tested with the following antibodies: CD9, CD81, CD63, TSG101, HSP70 and tubulin. Uncropped Western Blot image is available in Figure S1.

Isolated EVs were confirmed by transmission electron microscopy (TEM) and by Western blot analysis of selected EV markers in order to comply with the guidelines of the International Society of Extracellular Vesicles [31]. Results confirmed the presence of EVs within the expected range (30–100 nm), which were enriched for CD9, CD63 and CD81 markers (Figure 1). The detailed results of immunoblotting are shown in Figure S1.

By screening a 752-miRNA panel, the following 14 exo-oncomiRNAs were selected from the first phase study that were significantly altered after sTWEAK treatment, comparing either PC-3 or/and LNCaP cell lines, which accomplished the following criteria: cycle threshold (Ct) < 33 and at least >1.8-fold-over-expression when comparing both sTWEAK-stimulated cell lines (Table S1): miR-125b-1-3p, miR-193b-3p, miR-221-3p, miR-222-3p, miR-23a-3p, miR-27a-3p, miR-29a-3p, miR-31-5p, miR-497-5p, miR-643, miR-663b, miR-940, miR-9-5p and miR-99a-3p.

In the second phase of the experimental approach, we evaluated the expression levels of the 14 selected exo-oncomiRNAs in EVs isolated from liquid biopsy (semen and post-digital rectal examination urine) from 97 patients with low- or high-risk PCa. Pathological and clinical characteristics of patients are listed in Table 1. Gleason grade (GG) criteria and TNM classification was determined in accordance with the International Society of Urological Pathology (ISUP). Complementary examinations included prostate volume, measured by transrectal ultrasound, and PSA, as in standard clinical practice.

**Figure 2:**
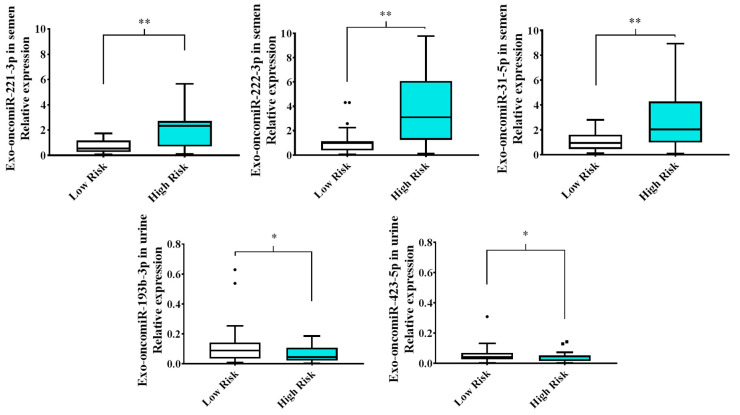
Exo-oncomiRNAs are differentially expressed in liquid biopsy from patients with prostate cancer. Box plots of relative expression of the 5 discriminatory exo-oncomiRNAs analyzed in semen and post-digital rectal examination urine liquid biopsies from patients with low- and high-risk PCa. Results are expressed as mean values ± SD. Statistical differences between groups are indicated: * *p* < 0.05; ** *p* < 0.01.

Analysis of the expression pattern of the 14 selected exo-oncomiRNAs in liquid biopsy of semen and post-digital rectal examination urine from patients with high-risk (ISUP Group III, IV and V) and low-risk (ISUP Group I and II) PCa revealed significant differences in the following five exo-oncomiRNAs: exo-oncomiR-221-3p, exo-oncomiR-222-3p, exo-oncomiR-31-5p, which were up-regulated in semen of high-risk patients versus low-risk patients; and exo-oncomiR-193-3p and exo-oncomiR-423-5p, which were down-regulated in post-digital rectal examination urine samples of high-risk patients (Figure 2). There were no significant differences between the studied groups for the remaining nine exo-oncomiRNAs (Table S2).

We also examined for clinical and metabolic differences between the high-risk and low-risk groups (Table 2). Univariate analysis showed that only total PSA was significantly higher in the high-risk group than in the low-risk group (*p* = 0.007), whereas sTWEAK semen levels were significantly lower in the high-risk group than in the low-risk group (*p* = 0.009) (Table 2), as has been reported [11]. We then tested for correlations between the five differentially expressed exo-oncomiRNAs and clinical and metabolic parameters using Spearman’s bivariate correlation coefficient test. The most relevant associations observed were the significant negative correlations between semen sTWEAK levels and the expression levels of exo-oncomiR-221-3p, exo-oncomiR-222-3p, and exo-oncomiR31-5p (r = −0.375 *p* = 0.017, r = −0.387 *p* = 0.013 and r = −0.364 *p* = 0.021, respectively) (Figure S2).

### 2.2. Semen Levels of Exo-oncomiR-221-3p May Help Identify an Aggressive Prostate Cancer Phenotype

**Figure 3:**
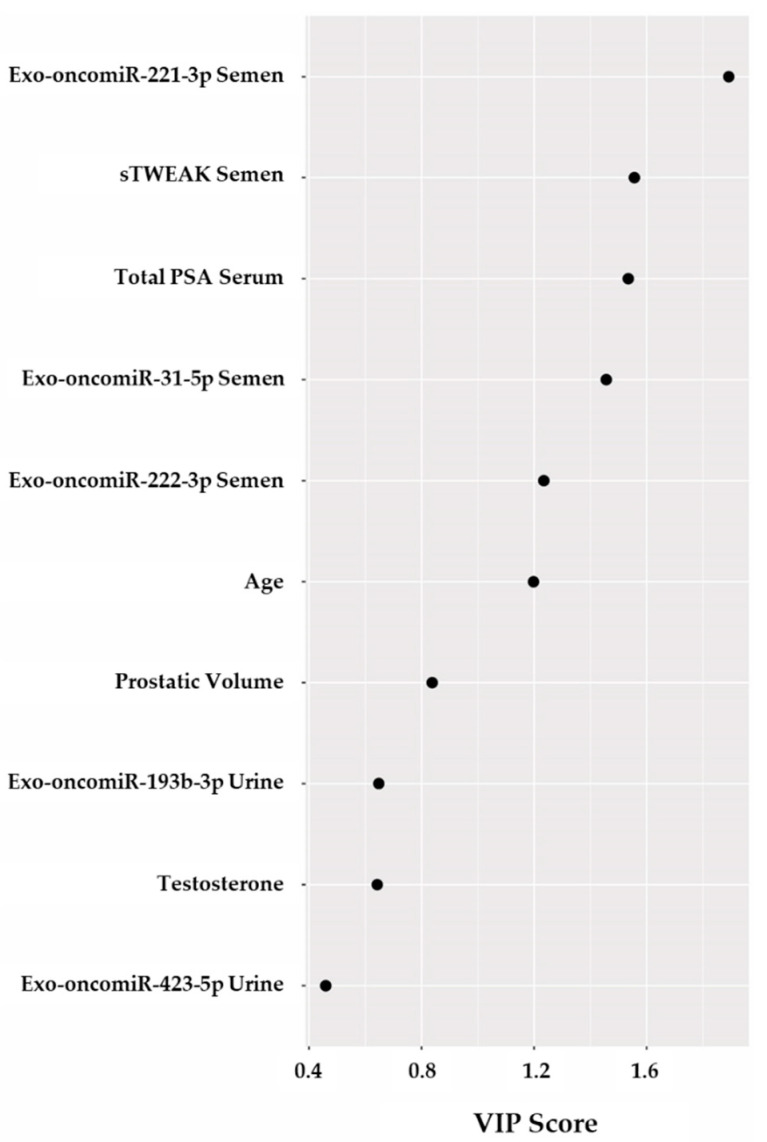
Variable importance in projection (VIP) scores. Selected variables: total PSA, testosterone, prostatic volume, age, sTWEAK in semen, exo-oncomiR-221-3p, exo-oncomiR222-3p, exo-oncomiR31-5p, exo-oncomiR-193-3p and exo-oncomiR-423-5p are shown in the model. Variables with scores close to or greater than 1 were considered important in the model.

We developed a partial least square-discriminant analysis (PLS-DA) model to evaluate the potential of the five selected exo-oncomiRNAs plus PSA in serum, sTWEAK in semen, age, prostatic volume, and testosterone, for the stratification of patients. Cross-validation analyses showed that a one-component model had an accuracy of 72.02% (R^2^ = 0.2073 and Q^2^ = 0.1493) (Figure S3a) indicating that is a good predictive model [32]. With regards to the importance of individual components, variable importance in projection (VIP) scores highlighted age, exo-oncomiR-222-3p in semen, exo-oncomiR-31-5p in semen, PSA in serum, sTWEAK in semen and exo-oncomiR-221-3p in semen as the most important variables (Figure 3). The VIP model estimated that exo-oncomiR-221-3p in semen and sTWEAK in semen had more influence than total PSA (Figure 3).

Variables with VIP score ≥ 1 were considered important in the model for determining PCa aggressiveness. To evaluate the usefulness of exo-oncomiRNAs as potential prognosis biomarkers of PCa aggressiveness in liquid biopsy, we performed logistic regression and receiver operating characteristic (ROC) curve analysis combining the following variables: exo-oncomiR-221-3p, exo-oncomiR-222-3p, exo-oncomiR-31-5p, total PSA, sTWEAK levels and age; Table 3 lists the different combinations. Results showed that the area under the curve (AUC) of each individual variable was below 0.8. Thus, we used a multivariate regression model combining each potential biomarker to test which combination was more suitable for correct diagnosis. Notably, we observed that the presence of exo-oncomiR-221-3p outperformed the other individual variables alone or in combination. The best panel in our study to distinguish PCa aggressiveness was that composed by exo-oncomiR-221-3p, exo-oncomiR-222-3p and semen sTWEAK, which could correctly classify 87.5% of patients, with an AUC of 0.857 and with 85.7% specificity and 76.9% sensitivity (Table 3) (Figure S3).

### 2.3. TWEAK Modulates Potential Predicted Targets for oncomiR-221-3p

**Figure 4:**
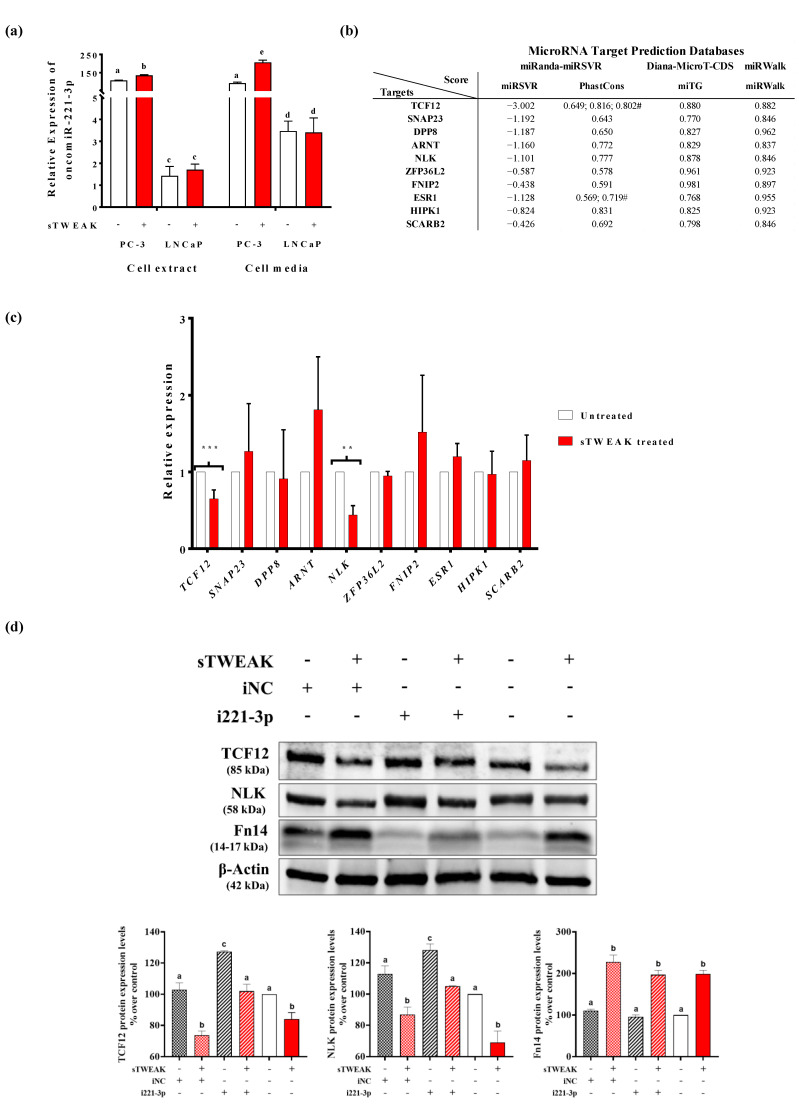
sTWEAK regulates oncomiR-221-3p expression and down-regulates NLK and TCF12 targets. (**a**) oncomiR-221-3p expression in PC-3 and LNCaP cell extracts and in extracellular vesicles (EVs) isolated from cell media. Different lettering over boxes indicates statistical differences. Significant differences are established at *p* < 0.05. Data are expressed as mean ± SEM (*n* = 4 experiments). (**b**) Selected targets for oncomiR-221-3p by 3 different target prediction algorithms. ^#^ conserved elements in multiply-aligned sequences. (**c**) qRT-PCR mRNA expression of selected oncomiR-221-3p targets in PC-3 cells before and after treatment with sTWEAK. Significant differences: ** *p* < 0.01; *** *p* < 0.001. Data are expressed as mean ± SEM (*n* = 6 experiments). Transcription factor 12 (TCF12); synaptosome associated protein 23 (SNAP23); dipeptidyl peptidase 8 (DPP8); aryl hydrocarbon receptor nuclear translocator (ARNT); Nemo-like kinase (NLK); ZFP36 ring finger protein-like 2 (ZFP36L2); folliculin interacting protein 2 (FNIP2); estrogen receptor 1 (ESR1); homeodomain interacting protein kinase 1 (HIPK1); and scavenger receptor class B-ember 2 (SCARB2). (**d**) Expression of TCF12, NLK and Fn14 protein in PC-3 cells transfected with the oncomiR-221-3p inhibitor and further treated with sTWEAK. Representative Western blots are presented (top). The membranes were tested with the corresponding antibody. iNC: inhibitor negative control, i221-3p: inhibitor miR-221-3p; Nemo-like kinase (NLK); transcription factor 12 (TCF12); fibroblast growth factor 14 (Fn14). Relative protein expression levels are shown (bottom), which were normalized to the corresponding control β-actin. Different lettering over boxes indicates statistical differences. Significant differences are established at *p* < 0.05. Data are expressed as mean ± SEM (*n* = 3 experiments). Full-length blots and gels are presented in Figure S4.

Several studies have shown that oncomiR-221 and oncomiR-222 are dysregulated in many cancers [33], including PCa [34,35], which is in line with our findings showing deregulated exo-oncomiR-221-3p and exo-oncomiR-222-3p in semen liquid biopsy of PCa. In vitro analysis showed that oncomiR-221-3p expression was found significantly up-regulated by sTWEAK only in PC-3 cells, both internally and in secreted EVs, and not in LNCaP cells, indicating that sTWEAK can potentially modulate oncomiR221-3p downstream targets (Figure 4a).

To demonstrate a direct effect of sTWEAK on oncomiR-221-3p targets, we first searched for possible oncomiR-221-3p targets and selected only those shared by the miRanda, Diana-MicroT-CDS and miRWalk databases. We obtained 69 genes with putative target sites for oncomiR-221-3p in their 3′untranslated regions. We then ranked the candidate genes by the miRSVR score (the lower the score, the stronger the match to the seed region); if two or more targets had a similar miRSVR score we considered the higher score from the Diana-microT-CDS algorithm, miTG. With these criteria, we selected 10 possible targets implicated in cancer and/or inflammation (Figure 4b, Table S3), shared also by onco-miR222-3p, because both miRNAS are encoded in tandem and contain identical seed sequences separated by 727 bases [33] (Table S4). Of the 10 targets only NLK (Nemo-like kinase) and TCF12 (transcription factor 12) expression levels were found to be reduced in PC-3 cells after sTWEAK treatment for 24 h by real-time PCR (Figure 4c) and Western blotting (Figure 4d). The stimulatory effect of sTWEAK was accompanied by the increased expression of its receptor Fn14 (Figure 4d).

Finally, we performed in vitro experiments using PC-3 cells and an oncomiR-221-3p inhibitor, which consistently influenced the expression of its target genes as demonstrated by the reduced expression of TCF12 and NLK proteins when compared with non-treated counterparts (Figure 4d). As anticipated, combined sTWEAK stimulation and oncomiR-221-3p inhibition resulted in a significant down-regulation of NLK and TCF12 protein levels (Figure 4d). The detailed results of immunoblotting are shown in Figure S4.

## 3. Discussion

Histopathological biopsy analysis is a common method for the diagnosis of PCa. This procedure, however, only enables the analysis of part of the prostatic gland and, because of the typical multifocal nature of PCa, information from a single biopsy is often insufficient and does not reflect the dynamics of the tumor in the prostate.

Diagnosis of cancer through the use of liquid biopsy has proven to be particularly useful as a non-invasive method of diagnosis and disease progression monitoring [36]. In a similar line, exosomal miRNAs isolated in the context of cancer, termed exo-oncomiRNAs, are promising biomarkers in part due to their stability in body fluids and ease of detection and quantification at low cost. Additionally, exo-oncomiRNAs have a very important role in modulating several critical cancer processes, including proliferation, migration, and angiogenesis, through their regulation of important target genes within the tumor environment [37].

Some exo-oncomiRNAs (e.g., miR-375, miR -21 and miR-141 [24,38]) from biofluids including blood and urine are known to have diagnostic and prognostic capacity in PCa. However, inconsistencies in identified, dysregulated exo-oncomiRNA profiles have been reported, likely due to a lack of standardized exosomal isolation and miRNA quantification techniques [23]. Despite these challenges, exo-oncomiRNAs remain highly promising biomarker candidates to aid in PCa diagnosis and prognosis.

We previously established a non-invasive biomarker panel with high negative predictive value to classify PCa aggressiveness. Specifically, this biofluid signature comprised the following biomarkers: total PSA serum levels, semen levels of sTWEAK, fasting serum glycemia, and mRNA expression levels of *Fn14*, *KLK2* (a gene that encodes a protease that activates pre-PSA) and two chemokine receptors (*CXCR2* and *CCR3*) in semen cell sediment. This panel can identify PCa aggressiveness with 90.9% success [11]. Although this panel could aid the clinical prognosis of PCa by outperforming the classical clinical biomarkers (age, T-classification, and total PSA serum levels), it requires the measurement of seven different biomarkers and uses two different biological samples—serum and semen.

In the aforementioned study, we observed that in patients with high-risk PCa, the decrease in sTWEAK levels in semen was accompanied by an increase in *Fn14* mRNA levels in seminal cell sediment, pointing to an active process of ligand–receptor interaction that may favor cell proliferation and migration, as described in PCa cell models [17,18]. Accordingly, the presence of TWEAK in PCa tumors could not only fuel tumor progression, but might also promote the secretion of exo-oncomiRNAs contained within EVs, which will likely have an impact on the tumor microenvironment.

In the search for an improved prognostic panel for PCa focusing on TWEAK-induced exo-oncomiRNAs, we show here that five exo-oncomiRNAs (exo-oncomiR-221-3p, exo-oncomiR-222-3p, exo-oncomiR-31-5p, exo-oncomiR-193b-3p, exo-oncomiR-423-5p) are significantly dysregulated between low- and high-risk PCa. VIP analysis of selected variables (including age, exo-oncomiRNA levels in semen and urine and, several analytical parameters) showed that variables with VIP scores greater than 1, considered of importance in the model for determining PCa aggressiveness, included only the three exo-oncomiRNAs expressed in semen (exo-oncomiR-221-3p, exo-oncomiR-222-3p and exo-oncomiR-31-5p). This finding may not be causal. Because 25% of semen is derived from prostatic tissue [39], its contents are more likely to contain prostate disease-specific exo-oncomiRNAs [29] than post-digital rectal examination urine samples [40].

After testing several logistic regression models followed by ROC analysis including the 3 selected biomarkers (exo-oncomiR-221-3p, exo-oncomiR-222-3p and sTWEAK), the measurements in semen liquid biopsy had the best prognostic accuracy (AUC = 0.857, *p* = 0.001) when compared with the ROC curve analysis using only serum PSA levels (AUC = 0.662, *p* < 0.007). This new model can outperform the classical PSA biomarker by 23.6% for a correct diagnosis, improving the classification efficacy up to 87.5%. If we include the two selected exo-oncomiRNAs (exo-oncomiR-221-3p, exo-oncomiR-222-3p) plus PSA levels in serum, the model can predict PCa severity better than is commonly reported by PSA screening alone; however, the model composed of sTWEAK, exo-oncomiR-221-3p and exo-oncomiR-222-3p—all measured in semen—improves not only the percentage of positively diagnosed patients by 2.25%, but increases the specificity by 8%.

MiR-221 and miR-222 are encoded tandemly in chromosome Xp11.3, and are highly homologous miRNAs sharing the same “seed sequences” [33,41]. In vivo studies have demonstrated that miR-221/222 down-regulation impairs the growth of PCa xenografts, pointing to miR-221-3p as an oncogenic miRNA in PCa [42]. In the present study, we observed that the addition of exo-oncomiR-221-3p expression levels in semen improves all prognostic model panel combinations. miR-221 is overexpressed in a variety of epithelial cancers including breast, liver, bladder, pancreas, gastric, colorectal cancer, melanoma, papillary thyroid carcinoma and glioblastoma [33]. Additionally, miR-221 has been found to be related to cancer progression in cervical squamous cell carcinoma [43], confers adriamycin resistance in breast cancer [44], and is a biomarker in hepatocellular carcinoma [45], diffuse large B cell lymphomas [46] and lung adenocarcinoma [47].

Studies on the expression of miR-221 in PCa (which is referred to as mir-221-3p in MirBase), have used only PCa tissue [48,49] and have found the levels to be up-regulated. Here we show, for the first time to our knowledge that the expression levels of miR-221-3p in PCa biofluids are higher in high-risk patients than in low-risk peers, and we additionally show that miR-221-3p is up-regulated in PC-3 secreted EVs and cell extracts. Mechanistically, in vitro studies have determined that miR-221-3p promotes proliferation of PCa cells [50].

MiR-221 directly targets NLK in neuroblastoma cells [51]. Accumulating evidence demonstrates that NLK has a pivotal role in cell proliferation, migration, invasion, and apoptosis by regulating a variety of transcriptional molecules [52]. NLK expression in PCa metastases is decreased in comparison with normal prostate epithelium and primary PCa [53]. Our findings show that oncomiR-221-3p inhibits NLK protein expression in PC-3 cells and that expression is further reduced by sTWEAK. An additional predicted and experimentally-demonstrated target for miR-221-3p is TCF12, a transcription factor member of the helix–loop–helix protein family found to be extensively expressed in many tissues [54]. As a target of miR-221, TCF12 has been related to survival after diagnosis of colon cancer [55], and there is evidence to suggest that TCF12 is involved in cell migration and differentiation [56]. Interestingly, the status of TCF12 has been found to be an independent predictor of biochemical recurrence-free survival in PCa [57]. We show here that miR-221-3p likely regulates TCF12 in PC-3 cells and its expression is, in turn, regulated by sTWEAK. While our findings point to the possibility that regulation of NLK or TCF12 might be a therapeutic approach against PCa tumors, further research and validation either in preclinical models or other established PCa cell lines will be needed to test their functional relevance in cell proliferation, invasion and chemosensitivity to cytotoxic agents.

Overall, our results reveal that TWEAK inflammation-induced exo-oncomiRNAs are components of an improved PCa prognostic panel based only on information obtained from a unique liquid biopsy, semen. Additionally, we reveal that a TWEAK inflammatory challenge in PCa cells can potentiate oncomiR-221-3p action.

## 4. Materials and Methods

### 4.1. Cell Culture

The PC-3 and LNCaP cell lines were purchased from Sigma-Aldrich (Barcelona, Spain). PC-3 cells were cultured in Ham’s F-12K (Kaighn’s) medium (1:1 mixture) with L-glutamate (Gibco, Fisher Scientific SL, Madrid, Spain), and LNCaP cells were cultured in RPMI 1640 medium supplemented with 1 mM sodium pyruvate (Gibco). Cultures were also supplemented with 10% fetal bovine serum, 1× antibiotic-antimycotic solution (Gibco), and 5 μg/mL plasmocin, and cultured in a humidified 5% CO_2_ atmosphere at 37 °C. Cells were grown in exosome-deprived serum overnight before stimulation for 24 h with 100 ng/mL human recombinant (hr) TWEAK (PeproTech, BioNova Cientifica, Barcelona, Spain).

### 4.2. Extracellular Vesicle Isolation from Cell Culture Media and Exo-oncomiRNA Expression Profile Using TaqMan Low-Density Arrays

Exosomes and other extracellular vesicles from cell culture media (PC-3 and LNCaP) were isolated and exo-oncomiRNAs were extracted using the exoRNeasy Serum/Plasma Maxi Kit (Qiagen, BioNova Cientifica, Madrid, Spain). For exo-oncomiRNA screening, the miRCURY LNA Universal RT microRNA PCR, Polyadenylation and cDNA Synthesis Kit (Exiqon, BioNova Cientifica, s.l. Madrid, Spain) was used for reverse transcription. cDNA was diluted and assayed by qRT-PCR according to the protocol in a 7900HT Fast Real-Time PCR System (Applied Biosystems, Thermo Fisher Scientic, Waltham, MA, USA). Each exo-oncomiRNA was assayed using ExiLENT SYBR Green Master Mix on the Human panel I+II, V5, miRCURY LNA miRNA miRNome PCR Panel (Qiagen) that included 752 mature human cancer-related miRNAs. Fluorescence readings and expression records of the microRNAs during the qRT-PCR were performed with the SDS 2.3 program (Applied Biosystems, Foster City, CA, USA). From the quantitative analysis by qRT-PCR of all miRNAs analyzed, we only considered those miRNAs that showed expression levels with a Ct < 33. Then, using the GeneGlobe program (Qiagen) [58], C_T_ values for each sample were normalized to the arithmetic mean of the following reference miRNAs, hsa-miR-423-5p, SNORD38B, SNORD49A, hsa-miR-191-5p, hsa-miR-103a-3p and U6 small nuclear RNA. The fold change expression of each exo-oncomiRNA was calculated with the formula 2^−ΔΔCt^ where each miRNA, regardless of the condition, was first normalized to the C_T_ of an endogenous control and then we calculated the ΔΔCt = ΔCt sample treated sTWEAK −ΔCt untreated controls. The exo-oncomiRNAs with *p* ≤ 0.05 when comparing cell type and condition and with an increase ≥1.8-fold were considered for further analysis.

### 4.3. Extracellular Vesicle Analysis

Extracellular vesicles from culture media, post-digital rectal examination urine and semen were obtained using exoRNeasy Serum/Plasma Maxi Kit just before miRNA isolation by the addition of 500 µL of elution buffer XE. The isolated EVs were further concentrated using a 100,000 Da cut-off concentrator (Amicon Ultra-0.5 mL Centrifugal Filters, Millipore). Samples were then ultrasonicated 3 times during 1 min bouts. Total protein was quantified using the BCA method (Pierce). A total amount of 10 μg EV protein and 10 μg total PC-3 cell extract were loaded on 4–15% SDS-PAGE gels and immunoblotted with polyclonal rabbit antibodies against: EXOAB-CD9A1, EXOAB-CD81A-1, EXOAB-CD63A-1, EXOABHsp70A-1, EXOAB-TSG101-1 (System Biology, Palo Alto, CA, USA), and the mouse monoclonal antibody for tubulin (Thermo Fisher Scientific, Waltham, MA, USA). HRP-conjugated goat anti-mouse or anti-rabbit (both from SBI) were used as secondary antibodies. All Western blots were developed with SuperSignal West Femto chemiluminescen substrate (Pierce Biotechnology, Boston, MA, USA) and visualized with the VersaDoc imaging system and Quantity One software (Bio-Rad, Barcelona, Spain) (Supplementary Materials).

### 4.4. Transmission Electron Microscopy Analysis

EVs were placed on carbon-coated copper grids (200 mesh), allowed to dry, and incubated in osmium tetroxide vapors for 15–30 min. TEM images were collected using a JEOL 1011 transmission electron microscope operating at 80 kV with a megaview III camera.

### 4.5. Patients

Our studied patient cohort comprised 97 consecutive patients with PCa who had undergone radical prostatectomy by open surgery at the University Hospital Joan XXIII, Tarragona, between 2015 and 2019—laparoscopic or robotic surgery (intraperitoneal or extraperitoneal—with or without bilateral ilio-obturator lymphadenectomy, according to the estimated risk of lymphadenopathy based on the Briganti nomogram [56]. Patients were stratified according to the 2014 ISUP-GG and TNM classification [57,58]. Patients were stratified into two categories: low-risk (ISUP Group I and II) and high-risk (ISUP Groups III, IV and V). Written informed consent prior to their inclusion was provided by all patients. The study was approved by our local ethics committee and performed according to the provisions of the Declaration of Helsinki (Biomedical Research Law 14/2007, Royal Decree of Biobanks 1716/2011, Organic Law15/1999 of September 13 Protection of Personal Data) [11]. Clinical parameters, tumor aggressiveness, and metabolic status of all patients were documented. All methods were approved and performed in accordance with guidelines and regulations of the Ethical Committee for Clinical Research (CEIm) from Pere Virgili Research Institute (Ref. CEim171/2017) (http://www.iispv.cat/plataformes_de_suport/en_comite-iispv.html). Patient’s inclusion criteria were as follows: older than 18 years, diagnosed with PCa by prostate biopsy in our center or any other, and treated by radical prostatectomy in our center. Exclusion criteria were patients with a previous history of cancer, patients older than 75 years, and those who had received any prior treatment before radical prostatectomy for PCa, as described [11].

### 4.6. Analytical Methods

Plasma glucose, cholesterol, triglyceride, high-density lipoprotein cholesterol, insulin levels and hepatic profile and renal profile was performed as described [59]. Levels of sTWEAK in semen were determined in duplicate using commercially available human enzyme-linked immunosorbent assay (ELISA) DuoSet Kits (R&D Systems Europe, Abingdon, UK).

### 4.7. Sample Processing

Serum/plasma: blood samples were collected after a fast of at least 12 h, or 2 h after an oral glucose tolerance test. Samples were centrifuged at 4 °C and stored at −80 °C.

Post-digital rectal examination urine: urine samples were collected prior to prostate biopsy or surgical intervention. Samples were centrifuged (2000× *g*, 10 min, 4 °C), and stored at −80 °C.

Semen: semen samples were centrifuged at 2000× *g* for 15 min at 22 °C to separate spermatozoa from semen plasma, and the supernatant (semen plasma) was stored at −80 °C.

All samples were processed and stored at the Institut d’Investigació Sanitària Pere Virgili (IISPV) BioBanc (B.0000853 + B.0000854) integrated in the Spanish National Biobanks Platform (PT13/0010/0029 and PT13/0010/0062) for its collaboration.

### 4.8. Extracellular Vesicles Extraction from Liquid Biopsy and Exo-onocomiRNA Quantitative Real-Time PCR Profiling

Extracellular vesicles and exo-miRNAs were isolated and extracted from urine and semen samples using the exoRNeasy Serum/Plasma Maxi Kit or Midi Kit (Qiagen) [12]. The miRCURY LNA Universal RT microRNA PCR, Polyadenylation and cDNA Synthesis Kit (Exiqon, BioNova Cientifica, s.l. Madrid, Spain) was used for reverse transcription. The expression profile of the 14 selected exo-oncomiRNAs was further analyzed in urine and semen samples in duplicate, using individual primers on a 7900HT Fast Real-Time PCR System (Applied Biosystems). Data were analyzed by SDS 2.3 and RQ Manager 1.2 (Applied Biosystems) using the 2^−ΔΔCt^ method. All values of Ct > 35 were excluded for further analysis.

### 4.9. Target Search by Bioinformatic Analysis

The targets of the selected exo-oncomiRNAs were searched using three target prediction software packages: (1) The miRanda algorithm (www.microRNA.org) was used to find potential target sites for miRNAs in the genomic sequence. From the miRanda algorithm results, we used the mirSVR score and PhastCons score to decipher which targets were potentially predicted. The mirSVR score is an estimate of the miRNA effect on the mRNA expression level; the more negative the score, the greater the inhibitory effect. PhastCons scores measure the conservation of nucleotide positions across vertebrates of any possible interaction; the higher the PhastCons value, the more conservative across vertebrates and the more important is the complementarity of the miRNA and the target [60]. (2) The Diana-MicroT-CDS predicts targets through the microT-CDS algorithm giving a miTG score, which is a general score for the predicted interaction. The closer the score is to 1, the greater the prediction confidence [61]. (3) Finally, we used the miRWalk platform. The calculated score is generated by executing the TarPmiR algorithm for miRNA target site prediction. The closer the score is to 1, the greater is the confidence prediction, in the same way as Diana-MicroT-CDS [62].

Candidate targets with an miR-SVR score equal or to less than −0.1; a PhastCons value equal or greater to 0.56 and, an miTG and miRWalk score equal or greater to 0.8 were considered as potential targets of exo-oncomiR-221-3p.

### 4.10. Functional Studies

Functional studies were performed in PC-3 cells cultured in 6-well plates and grown at 90% confluence. Cells were transfected using Lipofectamine 3000 (Invitrogen, Thermo Fisher Scientific, Waltham, MA, USA) and OPTI-MEM medium (Gibco) with a locked nucleic acid probe containing a specific sequence antisense oligonucleotide targeting exo-oncomiR-221-3p, miRCURY LNA exo-oncomiR-221-3p Power Inhibitor (Qiagen). A scrambled miRNA sequence, miRCURY LNA Power Inhibitor Control A (Qiagen), served as a negative control. Each condition was incubated for 24 h in a humidified 5% CO_2_ atmosphere at 37 °C before stimulation for 24 h with 100 ng/mL TWEAK (PeproTech) in serum-free media. After stimulation, cells were harvested for protein and RNA analysis. Expression analyses of target genes were performed using commercial individual primers on a 7900HT Fast Real-Time PCR System (Applied Biosystems). Protein analysis and Western blotting were performed using standard protocols. Nitrocellulose membranes were probed with the following primary antibodies that were purchased from Cell Signaling Technology (Danvers, MA, USA): TCF12/HEB (#11825) and NLK (#94350) and NF-κB2 p100 (#4882). An anti-β-actin (A11126) antibody was purchased from Sigma-Aldrich. The standard molecular weight marker used was purchased from New England Biolabs Inc. (Herts, UK). Western blots were developed with SuperSignal West Femto chemiluminescen substrate (Pierce Biotechnology, Boston, MA, USA) and visualized with VersaDoc imaging system and Quantity One software (Bio-Rad) (Supplementary Materials).

### 4.11. Statistical Analysis

For in vitro assays, experimental results are presented as mean ± standard error of the mean (SEM) of 3–4 experiments. Statistical significance was assessed with Student’s *t*-test. Results with *p* < 0.05 were considered statistically significant.

For human samples studies, the sample size was calculated to determine differences between exo-oncomiRNA expression levels in liquid biopsy with respect to the degree of aggressiveness of the tumor (low-risk/high-risk) in those patients diagnosed with PCa. We assumed a two-fold change difference between groups and identical standard deviation (SD) between the groups; therefore, a minimum of 35 patients was needed in each group (bilateral alpha error 0.05, power 90%). Statistical analysis were performed as described [11]. Briefly, for anthropometric and clinical variables, data are expressed as mean ± SD. Before statistical analysis, normal distribution was evaluated using Levene’s test. The non-parametric Mann–Whitney U-test was used to analyze the differences in anthropometric and clinical data and absolute expression levels of the exomiR candidates between patients according to ISUP-GG—low-risk (Group I and II) and high-risk (Group III, Group IV, and Group V). A *p*-value less than 0.05 was considered statistically significant. Spearman’s Rho test was used as a correlation analysis between anthropometric, clinical, and exo-miRNAs data. Partial least square discriminant analysis (PLS-DA) and VIP analysis models and binary logistic regression analysis were developed for selected variables. ROC curve analysis was performed to evaluate the best predictive model. The statistical software SPSS Statistics 24.0 (IBM, Madrid, Spain) package and R software (http://cran.r-project.org) were used for analysis.

## 5. Conclusions

TWEAK, exo-oncomiR -221-3p and exo-oncomiR-222-3p are proposed as an improved PCa prognostic panel based on information obtained from a unique biofluid, semen. Further studies in larger cohorts of PCa will be needed as a next step to confirm/validate our panel before it can be adopted in clinical practice.

## Figures and Tables

**Table 1:** Clinical and pathological characteristics of the studied cohort.

Patient’s Characteristics	Mean ± SD	*N*
**Age (years)**	63.5 ± 6.35	97
**Prostatic Volume (c.c)**	47.49 ± 23.09	97
**Testosterone (nmol/L)**	14.37 ± 5.07	97
**Total PSA (ng/mL)**	9.57 ± 7.92	97
		***N* (%)**
**BMI (kg/m^2^)**	<25	25 (25.8)
	25 ≤ x ≤ 29.99	50 (51.5)
	≥30	19 (19.6)
**Total PSA (ng/mL)**		
	<4	8 (8.2)
	4 ≤ x < 10	60 (61.9)
	≥ 10	29 (29.9)
**ISUP-GG**		
**Low Risk**	Group I	32 (33.0)
	Group II	25 (25.8)
**High Risk**	Group III	23 (23.7)
	Group IV	10 (10.3)
	Group V	7 (7.2)
**T pathological stage**		
	≤T2a	68 (70.1)
	T3,T4	29 (29.9)
**N pathological stage**		
	NX	57 (58.8)
	N0	34 (35.1)
	N1	6 (6.2)

Abbreviations: BMI, body mass index; ISUP-GG, International Society of Urological Pathology Gleason Grade groups based on the Gleason score as follows: (Gleason score ≤ 6—group I; 3 + 4 = 7 group II; 4 + 3 = 7 group III; 4 + 4 = 8—group IV; and 9–10—group V); PSA, prostate-specific antigen; T stage, Tumor category; N node, category. The bolded words differentiate the clinical and pathological characteristics from the rest of the table.

**Table 2:** Anthropometric and analytical characteristics according to ISUP-GG criteria.

	ISUP GG Classification		
Patient’s Stratification	Low-Risk	High-Risk	
	(Group I and II)	(Group III, IV and V)	
	*N* = 57	*N* = 40	
	Mean ± SD	Mean ± SD	*p*-Value
Anthropometric parameters			
Age (years)	62.46 ± 6.74	64.96 ± 5.52	0.066
BMI (kg/m^2^)	27.97 ± 4.07	27.64 ± 3.46	0.718
Prostatic volume (c.c)	48.68 ± 24.56	45.81 ± 21	0.687
Glycemic profile			
Glucose (mmol/L)	5.82 ± 1.1	6.29 ± 2.26	0.388
Insulin (pmol/L)	89.36 ± 58.39	87.42 ± 47.09	0.841
HOMA-IR	3.46 ± 2.58	3.67 ± 2.71	0.841
HbA1c (%)	5.74 ± 0.64	5.92 ± 0.84	0.364
Lipid profile			
Cholesterol (mmol/L)	5.03 ± 1.06	5.03 ± 1.1	0.957
HDL cholesterol (mmol/L)	1.49 ± 0.73	1.42 ± 0.39	0.672
LDL cholesterol (mmol/L)	3.28 ± 1.3	3 ± 0.88	0.503
Triglycerides (mmol/L)	1.36 ± 0.74	1.55 ± 0.96	0.711
Hepatic profile			
AST (µkat/L)	0.39 ± 0.19	0.33 ± 0.07	0.171
ALT (µkat/L)	0.42 ± 0.22	0.36 ± 0.11	0.402
GGT (µkat/L)	0.7 ± 0.85	0.65 ± 0.48	0.887
Renal profile			
Uric acid (µmol/L)	368.05 ± 83.1	456.2 ± 529.55	0.376
Urea (mmol/L)	14.26 ± 3.23	14.8 ± 5.12	0.808
Creatinine (μmol/L)	85.83 ± 18.44	80.05 ± 13.97	0.072
Hormonal profile			
SHBG (nmol/L)	46.13 ± 52.46	40.02 ± 16.36	0.814
Testosterone (nmol/L)	14.93 ± 4.62	13.55 ± 5.63	0.101
Tumoral marker			
Total PSA (μg/L)	7.71 ± 4.8	12.24 ± 10.43	0.007
Biofluid Biomarker profile			
Semen cytokines (pg/mg of total protein)			
sTWEAK	989.62 ± 685.75	617.25 ± 447.57	0.009
Exo-oncomiRNAs in semen—Relative expression levels			
miR-221-3p	0.75 ± 0.6	2.17 ± 1.7	0.002
miR-222-3p	2.01 ± 2.79	3.79 ± 2.92	0.006
miR-31-5p	1.05 ± 0.73	2.75 ± 2.27	0.004
Exo-oncomiRNAs in urine—Relative expression levels			
miR-193-3p	0.12 ± 0.12	0.06 ± 0.05	0.037
miR-423-5p	0.05 ± 0.05	0.04 ± 0.03	0.034

BMI, body mass index; HOMA-IR, homeostatic model assessment of insulin resistance; HbA1c, Hemoglobin A1c; HDL, high-density lipoprotein; LDL, low-density lipoprotein; AST, aspartate aminotransferase; ALT, alanine aminotransferase; GGT, γ-Glutamyltransferase; SHBG, sex hormone-binding globulin; PSA, prostate-specific antigen; sTWEAK, soluble tumor necrosis factor-like weak inducer of apoptosis.

**Table 3:** Exo-oncomiRNAs-based models as diagnostic classifiers.

				95% CI				
ROC Model	AUC	Error	*p*-Value	Lower	Upper	Sensivity (%)	Specificity (%)	% Correct Diagnosis
**Age**	0.610	0.058	0.066	0.496	0.724	85	75.4	62.9
**Total PSA**	0.662	0.056	0.007	0.552	0.772	85	31.6	63.9
**sTWEAK**	0.708	0.072	0.009	0.567	0.848	85.7	52.8	71.9
**exo-oncomiR-221-3p**	0.79	0.078	0.002	0.638	0.943	86.7	55.6	78.6
**exo-oncomiR-222-3p**	0.758	0.08	0.006	0.601	0.915	86.7	74.1	66.7
**exo-oncomiR-31-5p**	0.768	0.082	0.004	0.607	0.929	86.7	48.1	76.2
**Total PSA + Age**	0.704	0.054	0.001	0.597	0.810	85	70.2	67
**Total PSA + sTWEAK**	0.738	0.072	0.003	0.597	0.879	85.7	47.2	71.9
**Total PSA + exo-oncomiR-221-3p**	0.864	0.063	<0.001	0.74	0.998	86.7	55.6	83.3
**Total PSA + exo-oncomiR-222-3p**	0.78	0.071	0.003	0.641	0.919	86.7	55.6	73.8
**Total PSA + exo-oncomiR-31-5p**	0.832	0.07	<0.001	0.695	0.969	86.7	51.9	81
**sTWEAK + Age**	0.709	0.069	0.009	0.574	0.844	85.7	50	66.7
**sTWEAK + exo-oncomiR-221-3p**	0.841	0.073	<0.001	0.698	0.983	85.7	69.2	82.5
**sTWEAK + exo-oncomiR-222-3p**	0.745	0.086	0.012	0.576	0.913	85.7	42.3	70
**sTWEAK + exo-oncomiR-31-5p**	0.808	0.077	0.001	0.657	0.958	85.7	61.5	77.5
**exo-oncomiR-221-3p + Age**	0.802	0.077	0.001	0.651	0.954	86.7	33.3	76.2
**exo-oncomiR-221-3p + exo-oncomiR-222-3p**	0.802	0.078	0.001	0.65	0.955	86.7	63	76.2
**exo-oncomiR-221-3p + exo-oncomiR-31-5p**	0.8	0.079	0.001	0.646	0.954	86.7	55.6	81
**exo-oncomiR-222-3p + Age**	0.751	0.081	0.008	0.592	0.909	86.7	66.7	73.8
**exo-oncomiR-222-3p + exo-oncomiR-31-5p**	0.8	0.077	0.001	0.649	0.951	86.7	55.6	81
**exo-oncomiR-31-5p + Age**	0.778	0.078	0.003	0.625	0.930	86.7	44.4	73.8
**Total PSA + sTWEAK + Age**	0.746	0.067	0.002	0.614	0.878	85.7	44.4	73.7
**Total PSA + sTWEAK + exo-oncomiR-221-3p**	0.863	0.068	<0.001	0.73	0.996	85.7	69.2	85
**Total PSA + sTWEAK + exo-oncomiR-222-3p**	0.758	0.086	0.008	0.59	0.926	85.7	46.2	75
**Total PSA + sTWEAK + exo-oncomiR-31-5p**	0.824	0.076	0.001	0.675	0.974	85.7	73.1	77.5
**Total PSA + exo-oncomiR-221-3p + Age**	0.889	0.056	<0.001	0.780	0.998	85.7	37	83.3
**Total PSA + exo-oncomiR-221-3p + exo-oncomiR-222-3p**	0.872	0.06	<0.001	0.755	0.988	86.7	59.3	83.3
**Total PSA + exo-oncomiR-221-3p + exo-oncomiR-31-5p**	0.854	0.067	<0.001	0.724	0.985	86.7	51.9	83.3
**Total PSA + exo-oncomiR-222-3p + Age**	0.840	0.064	<0.001	0.714	0.965	86.7	37	83.3
**Total PSA + exo-oncomiR-222-3p + exo-oncomiR-31-5p**	0.849	0.069	<0.001	0.713	0.985	86.7	59.3	83.3
**Total PSA + exo-oncomiR-31-5p + Age**	0.862	0.061	<0.001	0.743	0.981	86.7	37	83.3
**sTWEAK + exo-oncomiR-221-3p + Age**	0.854	0.067	<0.001	0.723	0.986	85.7	23.7	77.5
**sTWEAK + exo-oncomiR-221-3p + exo-oncomiR-222-3p**	0.857	0.069	<0.001	0.721	0.993	85.7	76.9	87.5
**sTWEAK + exo-oncomiR-221-3p + exo-oncomiR-31-5p**	0.841	0.073	<0.001	0.698	0.983	85.7	69.2	82.5
**sTWEAK + exo-oncomiR-222-3p + Age**	0.764	0.078	0.006	0.611	0.917	85.7	50	72.5
**sTWEAK + exo-oncomiR-222-3p + exo-oncomiR-31-5p**	0.83	0.073	0.001	0.687	0.972	85.7	53.8	82.5
**sTWEAK + exo-oncomiR-31-5p + Age**	0.821	0.074	0.001	0.677	0.966	85.7	34.6	75
**exo-oncomiR-221-3p + exo-oncomiR-222-3p + exo-oncomiR-31-5p**	0.807	0.076	0.001	0.658	0.956	86.7	51.9	83.3
**exo-oncomiR-221-3p + exo-oncomiR-222-3p + Age**	0.820	0.074	0.001	0.675	0.965	86.7	25.9	76.2
**exo-oncomiR-221-3p + exo-oncomiR-31-5p + Age**	0.812	0.074	0.001	0.668	0.957	86.7	37	78.6
**exo-oncomiR-222-3p + exo-oncomiR-31-5p + Age**	0.802	0.075	0.001	0.655	0.950	86.7	44	78.6
**Total PSA + sTWEAK + exo-oncomiR-221-3p + exo-oncomiR-222-3p**	0.86	0.071	<0.001	0.721	0.999	85.7	69.2	85
**Total PSA + sTWEAK + exo-oncomiR-221-3p + exo-oncomiR-31-5p**	0.86	0.069	<0.001	0.724	0.995	85.7	69.2	85
**Total PSA + sTWEAK + exo-oncomiR-222-3p + exo-oncomiR-31-5p**	0.83	0.076	0.001	0.682	0.978	85.7	65.4	82.5
**Age + Total PSA + sTWEAK + exo-oncomiR-221-3p**	0.879	0.62	<0.001	0.757	1	85.7	23.1	85
**Age + Total PSA + sTWEAK + exo-oncomiR-222-3p**	0.808	0.074	0.001	0.662	0.953	85.7	50	82.5
**Age + Total PSA + sTWEAK + exo-oncomiR-31-5p**	0.849	0.069	<0.001	0.715	0.983	85.7	53.2	82.5
**Age + Total PSA + exo-oncomiR-221-3p + exo-oncomiR-222-3p**	0.894	0.053	<0.001	0.789	0.998	86.7	56.7	83.3
**Age + Total PSA + exo-oncomiR-221-3p + exo-oncomiR-31-5p**	0.879	0.059	<0.001	0.764	0.994	86.7	54.3	83.3
**Age + Total PSA + exo-oncomiR-222-3p + exo-oncomiR-31-5p**	0.867	0.059	<0.001	0.752	0.982	86.7	49.2	81
**Age + sTWEAK + exo-oncomiR-221-3p + exo-oncomiR-222-3p**	0.868	0.061	<0.001	0.748	0.988	86.7	46.5	80
**Age + sTWEAK + exo-onxomiR-221-3p + exo-oncomiR-31-5p**	0.857	0.067	<0.001	0.726	0.988	85.7	76.9	80
**Age + sTWEAK + exo-oncomiR-222-3p + exo-oncomiR-31-5p**	0.832	0.070	0.001	0.695	0.969	86.7	46.5	80
**Age + exo-oncomiR-221-3p + exo-oncomiR-222-3p + exo-oncomiR-31-5p**	0.820	0.072	0.001	0.678	0.962	86.7	48.1	81
**Total PSA + exo-oncomiR-221-3p + exo-oncomiR-222-3p + exo-oncomiR-31-5p**	0.874	0.061	<0.001	0.754	0.995	86.7	55.6	83.3
**sTWEAK + exo-oncomiR-221-3p + exo-oncomiR-222-3p + exo-oncomiR-31-5p**	0.86	0.068	<0.001	0.726	0.994	85.7	73.1	85
**Total PSA + sTWEAK + exo-oncomiR-221-3p + exo-oncomiR-222-3p + exo-oncomiR-31-5p**	0.865	0.069	<0.001	0.73	1	85.7	69.2	85
**Age + Total PSA + sTWEAK + exo-oncomiR-221-3p + exo-oncomiR-222-3p**	0.879	0.062	<0.001	0.757	1	86.7	70.4	87.5
**Age + Total PSA + sTWEAK + exo-oncomiR-221-3p + exo-oncomiR-31-5p**	0.879	0.062	<0.001	0.758	1	86.7	57.9	85
**Age + Total PSA + sTWEAK + exo-oncomiR-222-3p + exo-oncomiR-31-5p**	0.857	0.065	<0.001	0.729	0.985	85.7	58.3	85
**Age + Total PSA + exo-oncomiR-221-3p + exo-oncomiR-222-3p + exo-oncomiR-31-5p**	0.896	0.053	<0.001	0.793	0.999	86.7	57.9	83.3
**Age + sTWEAK + exo-oncomiR-221-3p + exo-oncomiR-222-3p + exo-oncomiR-31-5p**	0.874	0.061	<0.001	0.755	0.992	85.7	63.8	82.5
**Age + Total PSA + sTWEAK + exo-oncomiR-221-3p + exo-oncomiR-222-3p + exo-oncomiR-31-5p**	0.879	0.062	<0.001	0.757	1	85.7	68.9	87.5

Receiver operating characteristic (ROC) curve values showing the predictive efficiency for distinguishing PCa aggressiveness. Percentage of correct diagnostic values was obtained by multivariate models (backward stepwise, conditional method). AUC, area under the curve; 95% CI (confidence interval). The bolded words represent the ROC Models.

## Data Availability

The data presented in this study is contained within the article and the supplementary material.

## Supplementary Materials

The following are available online at https://www.mdpi.com/2072-6694/13/2/250/s1. Table S1: 14 identified exo-oncomiRNAS, Table S2: Exo-oncomiRNAS non-significantly changed when comparing urine or semen biofluids from patients with PCa stratified by risk (low or high), Table S3: Role of elected exo-miR-221-3p targets in cancer, Table S4: List of the selected exo-oncomiR-221-3p and exo-oncomiR-222-3p target’s scores, Figure S1: Complete Western blot (WB) results referring to Figure 1b, Figure S2: Spearman’s correlation, Figure S3a,b: PLS-DA analysis, Figure S4: Complete WB results referring to Figure 4d.
