# Supplementary material for: Liquid Biopsy-Based Exo-oncomiRNAs Can Predict Prostate Cancer Aggressiveness
Source: Cancers (Basel). 2021 Jan 11;13(2):250. doi: 10.3390/cancers13020250 (PMC7826893; doi:10.3390/cancers13020250)
Supplement: Supplementary file 1 [file cancers-13-00250-s001.pdf]

Supplementary Material: Liquid Biopsy-Based Exo-oncomiRNAs Can Predict Prostate Cancer Aggressiveness

Xavier Ruiz-Plazas,Antonio Altuna-Coy, Marta Alves-Santiago, José Vila-Barja, Joan Francesc García-Fontgivell, Salomé Martínez-González, José Segarra-Tomás and Matilde R. Chacón

Table S1. List of the 14 selected exo-oncomiRNAs and fold-change that accomplished the following criteria: Ct < 33 and > 1.8 fold over- expression when comparing PC-3 and/or LNCaP cell lines treated with sTWEAK.

	Fold Change		
**Exo-oncomiRNA**	**PC-3 vs PC-3 + sTWEAK**	**LNCaP vs LNCaP + sTWEAK**	**PC-3 + sTWEAK vs LNCaP + sTWEAK**
miR-125b-1-3p	-	1.83	-
miR-193b-3p	-	-	14.03
miR-221-3p	3.87	-	-
miR-222-3p	2.05	-	-
miR-23a-3p	-	-	5.24
miR-27a-3p	-	2.25	-
miR-29a-3p	-	-	2.14
miR-31-5p	1.82	-	-
miR-497-5p	-	-	3.67
miR-643	2.96	-	-
miR-663b	-	2.27	-
miR-940	2.11	-	-
miR-9-5p	-	2.81	-
miR-99a-3p	-	2.95	-

Table S2. List of the 9 out of 14 selected exo-oncomiRNAs that did not show differences when comparing urine or semen biofluids from PCa patients stratified by risk (low or high).

	ISUP-GG Classification						
	**Low-Risk (Group I and II) *N* = 57**			**High-Risk (Group III, IV & V) *N* = 40**			
	**Mean**	**±**	**S.D**	**Mean**	**±**	**S.D**	***p*-Value**
Exo-oncomiRNAs in semen - Relative expression levels							
miR-125b-2-3p	1.49	±	0.78	1.99	±	1.58	0.408
miR-193b-3p	0.99	±	0.54	0.99	±	0.42	0.723
miR-23a-3p	1.54	±	0.69	2.15	±	3.54	0.439
miR-27a-3p	1.66	±	1.02	1.44	±	0.80	0.487
miR-29a-5p	2.05	±	1.29	1.98	±	1.32	0.865
miR-497-5p	0.99	±	0.58	0.91	±	0.34	0.844
miR-643	0.44	±	0.39	0.63	±	0.52	0.185
miR-663b	1.68	±	2.52	2.68	±	4.68	0.865
miR-9-5p	0.98	±	0.80	2.34	±	2.83	0.131
miR-940	1.40	±	2.41	2.46	±	2.89	0.265
miR-99a-3p	1.25	±	0.66	1.27	±	0.52	0.762
Exo-oncomiRNAs in urine - Relative expression levels							
miR-125b-2-3p	0.05	±	0.06	0.04	±	0.03	0.549
miR-221-3p	0.03	±	0.03	0.02	±	0.01	0.555
miR-222-3p	0.03	±	0.04	0.02	±	0.03	0.838
miR-23a-3p	0.07	±	0.04	0.06	±	0.03	0.404
miR-29a-3p	0.03	±	0.04	0.02	±	0.03	0.085
miR-31-5p	0.02	±	0.01	0.02	±	0.02	0.222
miR-497-5p	0.02	±	0.03	0.02	±	0.02	0.476
miR-643	0.30	±	1.12	0.13	±	0.28	0.716
miR-663b	0.04	±	0.08	0.02	±	0.04	0.500
miR-9-5p	0.12	±	0.16	0.16	±	0.28	0.583

miR-940	0.10	±	0.12	0.12	±	0.15	0.640
miR-99a-3p	0.03	±	0.05	0.02	±	0.01	0.713

Table S3. Predicted miR-221-3p targets.

Target Gene Symbol	Gene Name	Biological Function	Role Associated with Cancer
TCF12	Transcription Factor 12	May participate in regulating lineage-specific gene expression through the formation of heterodimers	Colorectal cancer survival [1]<br>Ovarian cancer cell proliferation, migration and invasion [2]<br>High expression contributes to gastric cancer development [3]<br>Decreased expression associated with PCa biochemical recurrence [4]
SNAP23	Synaptosome Associated Protein 23	Regulator of transport vesicle docking and fusion	Supresses cervical cancer progression [5]<br>Hiperexpression promotes ovarian cancer [6].
DPP8	Dipeptidyl Peptidase 8	May play a role in T-cell activation and immune function	Inhibition induces cell death in multiple myeloma [7]<br>Expression may be related to less aggressive disease in advanced-stage ovarian cancer [8]
ARNT	Aryl Hydrocarbon Receptor Nuclear Translocator	The protein is a co-factor for transcriptional regulation by hypoxia-inducible factor 1	Alters gene expression of tumor growth-related protein COX-2 in squamous cell carcinoma [9]<br>Increased activity promotes tumor progression [10]
NLK	Nemo Like Kinase	Serine/threonine-protein kinase that regulates a number of transcription factors with key roles in cell fate determination.	mRNA and protein expression was significantly increased in laryngeal carcinoma tissues compared with those in adjacent tissues [11]<br>Inhibits androgen receptor signaling in PCa cells [12]
ZFP36L2	ZFP36 Ring Finger Protein Like 2	Nuclear transcription factor most likely functions in regulating the response to growth factors	Promotes pancreatic cancer aggressiveness[13]
FNIP2	Folliculin Interacting Protein 2	May play a role cellular metabolism and nutrient sensing by regulating the AMPK-mechanistic target	Possible role in Kidney tumor progression [14]<br>Somatic mutation associated with gastric and colon cancers [15]
ESR1	Estrogen Receptor 1	Essential for sexual development and reproductive function	Mutation are associated with advanced breast cancer [16]
HIPK1	Homeodomain Interacting Protein Kinase 1	Phosphorylates homeodomain transcription factors and may also function as a co-repressor for homeodomain transcription factors	Down regulation in human colorectal adenocarcinoma cells modulates resistance to Methotrexate [17]<br>Regulation of breast cancer stem cell Heterogeneity via targeting HIPK1/beta-Catenin Axis [18]
SCARB2	Scavenger Receptor Class B Member 2	May participate in membrane transportation and the reorganization of endosomal/lysosomal compartment	Role in the regulation of intracellular vesicle trafficking of lysosomes and endosomes in human breast cancer cells [19]

References

1. Mullany, L.E.; Herrick, J.S.; Wolff, R.K.; Stevens, J.R.; Samowitz, W.; Slattery, M.L. Transcription factor-microRNA associations and their impact on colorectal cancer survival. *Mol. Carcinog.* **2017**, *56*, 2512–2526, doi:10.1002/mc.22698.
2. Gao, S.; Bian, T.; Su, M.; Liu, Y.; Zhang, Y. miR-26a inhibits ovarian cancer cell proliferation, migration and invasion by targeting TCF12. *Oncol. Rep.* **2019**, *43*, 368–374, doi:10.3892/or.2019.7417.
3. Wang, X.; Gao, S.; Xie, F.; Li, W.; Li, M.; Yan, N.; Gao, T.; Fang, L. High expression of TCF12 contributes to gastric cancer development via being target regulated by miR-183 and activating PI3K/AKT pathway. *J. Cell. Biochem.* **2019**, *120*, 13903–13911, doi:10.1002/jcb.28664.
4. Chen, Q.-B.; Liang, Y.-K.; Zhang, Y.-Q.; Jiang, M.-Y.; Han, Z.-D.; Liang, Y.-X.; Wan, Y.-P.; Ying-Ke, L.; He, H.-C.; Zhong, W.-D. Decreased expression of TCF12 contributes to progression and predicts biochemical recurrence in patients with prostate cancer. *Tumor Biol.* **2017**, *39*, doi:10.1177/1010428317703924.
5. Zhu, B.; Zhang, Q.; Wu, Y.; Luo, J.; Zheng, X.; Xu, L.; Lu, E.; Qu, J.; Ren, B. SNAP23 suppresses cervical cancer progression via modulating the cell cycle. *Gene* **2018**, *673*, 217–224, doi:10.1016/j.gene.2018.06.028.

6. Sun, Q.; Huang, X.; Zhang, Q.; Qu, J.; Shen, Y.; Wang, X.; Sun, H.; Wang, J.; Xu, L.; Chen, X.; et al. SNAP23 promotes the malignant process of ovarian cancer. *J. Ovarian Res.* **2016**, *9*, 1–9, doi:10.1186/s13048-016-0289-9.
7. Sato, T.; Tatekoshi, A.; Takada, K.; Iyama, S.; Kamihara, Y.; Jawaid, P.; Rehman, M.U.; Noguchi, K.; Kondo, T.; Kajikawa, S.; et al. DPP8 is a novel therapeutic target for multiple myeloma. *Sci. Rep.* **2019**, *9*, 1–8, doi:10.1038/s41598-019-54695-w.
8. Brunetti, M.; Holth, A.; Panagopoulos, I.; Staff, A.C.; Micci, F.; Davidson, B. Expression and clinical role of the dipeptidyl peptidases DPP8 and DPP9 in ovarian carcinoma. *Virchows Archiv* **2018**, *474*, 177–185, doi:10.1007/s00428-018-2487-x.
9. Chang, K.-Y.; Shen, M.-R.; Lee, M.-Y.; Wang, W.-L.; Su, W.-C.; Chang, W.-C.; Chen, B.-K. Epidermal Growth Factor-activated Aryl Hydrocarbon Receptor Nuclear Translocator/HIF-1β Signal Pathway Up-regulates Cyclooxygenase-2 Gene Expression Associated with Squamous Cell Carcinoma. *J. Biol. Chem.* **2009**, *284*, 9908–9916, doi:10.1074/jbc.m806210200.
10. Semenza, G.L. HIF-1 and tumor progression: pathophysiology and therapeutics. *Trends Mol. Med.* **2002**, *8*, S62–S67, doi:10.1016/s1471-4914(02)02317-1.
11. Shen, N.; Duan, X.-H.; Wang, X.-L.; Yang, Q.-Y.; Feng, Y.; Zhang, J.-X. Effect of NLK on the proliferation and invasion of laryngeal carcinoma cells by regulating CDCP1. *Eur. Rev. Med. Pharmacol. Sci* 2019, *23*, 6226–6233.
12. Emami, K.H.; Brown, L.G.; Pitts, T.E.; Sun, X.; Vessella, R.L.; Corey, E. Nemo-like kinase induces apoptosis and inhibits androgen receptor signaling in prostate cancer cells. *Prostate* **2009**, *69*, 1481–1492, doi:10.1002/pros.20998.
13. Yonemori, K.; Seki, N.; Kurahara, H.; Osako, Y.; Idichi, T.; Arai, T.; Koshizuka, K.; Kita, Y.; Maemura, K.; Natsugoe, S. ZFP36L2promotes cancer cell aggressiveness and is regulated by antitumormicroRNA-375in pancreatic ductal adenocarcinoma. *Cancer Sci.* **2017**, *108*, 124–135, doi:10.1111/cas.13119.
14. Hasumi, H.; Baba, M.; Hasumi, Y.; Lang, M.; Huang, Y.; Oh, H.F.; Matsuo, M.; Merino, M.J.; Yao, M.; Ito, Y.; et al. Folliculin-interacting proteins Fnip1 and Fnip2 play critical roles in kidney tumor suppression in cooperation with Flcn. *Proc. Natl. Acad. Sci.* **2015**, *112*, E1624–E1631, doi:10.1073/pnas.1419502112.
15. Mo, H.Y.; Son, H.J.; Choi, E.J.; Yoo, N.J.; An, C.-H.; Lee, S.H. Somatic mutations of candidate tumor suppressor genes folliculin-interacting proteins FNIP1 and FNIP2 in gastric and colon cancers. *Pathol. - Res. Pr.* **2019**, *215*, 152646, doi:10.1016/j.prp.2019.152646.
16. Lee, N.; Park, M.-J.; Song, W.; Jeon, K.; Jeong, S. Currently Applied Molecular Assays for Identifying *ESR1* Mutations in Patients with Advanced Breast Cancer. *Int. J. Mol. Sci.* **2020**, *21*, 8807, doi:10.3390/ijms21228807.
17. Zhang, D.; Li, Y.; Sun, P. miR-770-5p modulates resistance to methotrexate in human colorectal adenocarcinoma cells by downregulating HIPK1. *Exp. Ther. Med.* **2019**, *19*, 339–346, doi:10.3892/etm.2019.8221.
18. Liu, B.; Du, R.; Zhou, L.; Xu, J.; Chen, S.; Chen, J.; Yang, X.; Liu, D.-X.; Shao, Z.-M.; Zhang, L.; et al. miR-200c/141 Regulates Breast Cancer Stem Cell Heterogeneity via Targeting HIPK1/β-Catenin Axis. *Theranostics* **2018**, *8*, 5801–5813, doi:10.7150/thno.29380.
19. Nishimura, Y.; Yoshioka, K.; Bernard, O.; Himeno, M.; Itoh, K. LIM kinase 1: evidence for a role in the regulation of intracellular vesicle trafficking of lysosomes and endosomes in human breast cancer cells. *Eur. J. Cell Biol.* **2004**, *83*, 369–380, doi:10.1078/0171-9335-00382.

Table S4. List of the selected exo-oncomiR-221-3p and exo-oncomiR-222-3p target's scores.

MicroRNA Target Prediction Databases								
	miRanda-miRSVR				Diana-MicroT-CDS		miRWalk	
Score	miRSVR		PhastCons		miTG		miRWalk	
Targets	exo-miR-221-3p	exo-miR-222-3p	exo-miR-221-3p	exo-miR-222-3p	exo-miR-221-3p	exo-miR-222-3p	exo-miR-221-3p	exo-miR-222-3p
**TCF12**	-3.002	-2.370	0.649; 0.816; 0.802#	0.649; 0.816; 0.802#	0.880	0,861	0.882	0,807
**SNAP23**	-1.192	-1.191	0.643	0,648	0.770	0,789	0.846	0,801
**DPP8**	-1.187	-1.181	0.650	0,650	0.827	0,805	0.962	0,923
**ARNT**	-1.160	-1.172	0.772	0,772	0.829	0,836	0.837	0,923
**NLK**	-1.101	-1.099	0.777	0,777	0.878	0,868	0.846	0,823
**ZFP36L2**	-0.587	-0,608	0.578	0,578	0.961	0,956	0.923	0,846
**FNIP2**	-0.438	-0,404	0.591	0,586	0.981	0,947	0.897	1,000
**ESR1**	-1.128	-0,844	0.569; 0.719#	0,719	0.768	0,702	0.955	1,000
**HIPK1**	-0.824	-0,851	0.831	0,831	0.825	0,956	0.923	0,923
**SCARB2**	-0.426	-0,429	0.692	0,692	0.798	0,829	0.846	0,846

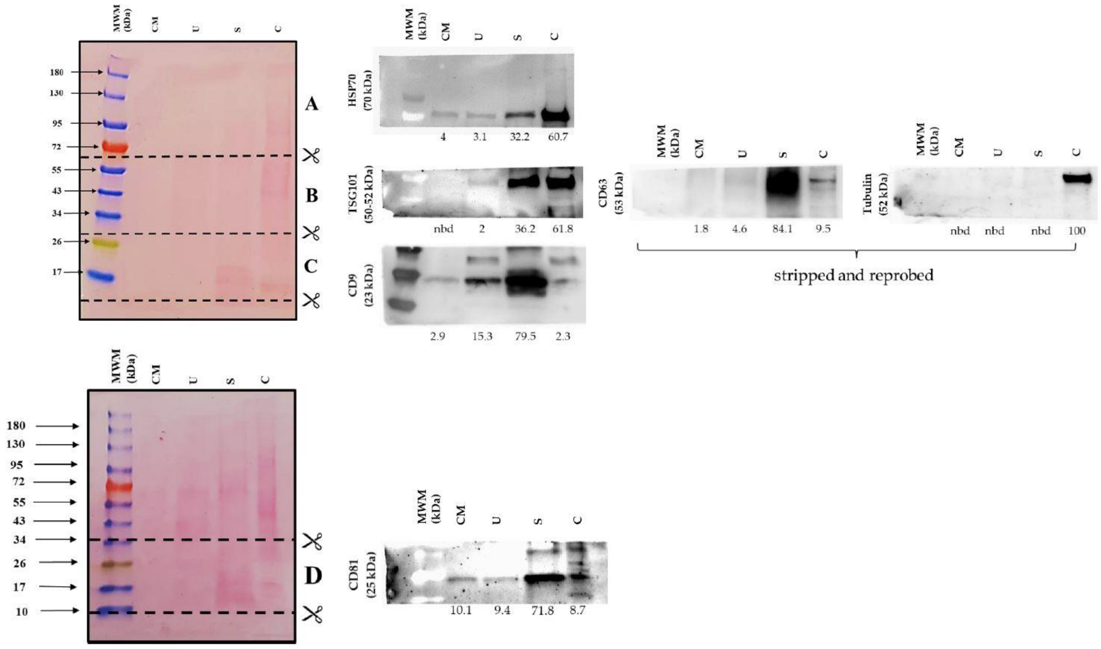

Figure S1. Complete WB results referring to Figure 1. Ponceau stained images of gel transferred-membranes cut before antibody incubation to allow detection of CD9, TSG101, HSP70 proteins (A,B,C). Then stripping off was performed and membranes were reproved with CD63 and Tubulin antibodies (D). A separate gel was run and incubated with CD81 antibody. The numbers below the membranes represent the percentage of intensity. CM: culture media, U: urine, S: semen, C: PC-3 cell extract nbd: no band detected.

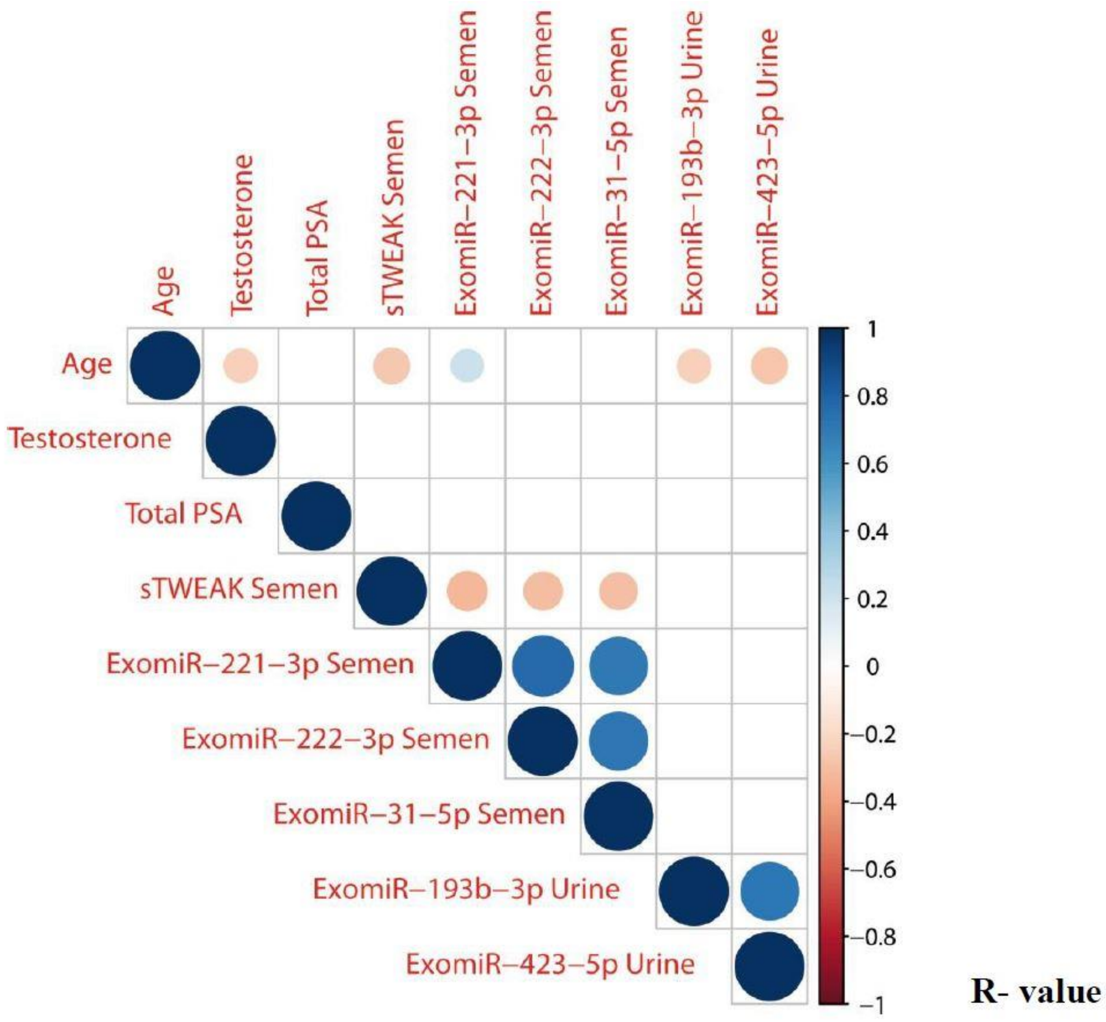

Figure S2. Spearman correlation matrix. Correlation map plotted using significance levels for Spearman´s test performed with relevant clinical and biomarker data from all studied patients. Positive correlations are displayed in grading-blue and negative correlations in grading-red color. Correlations with *p*-value ≥0.05 are considered as insignificant and are left blank. Color intensity and the size of the circle are proportional to the correlation coefficients. In the right side of the correlogram, the legend color shows the correlation coefficients and the corresponding colors.

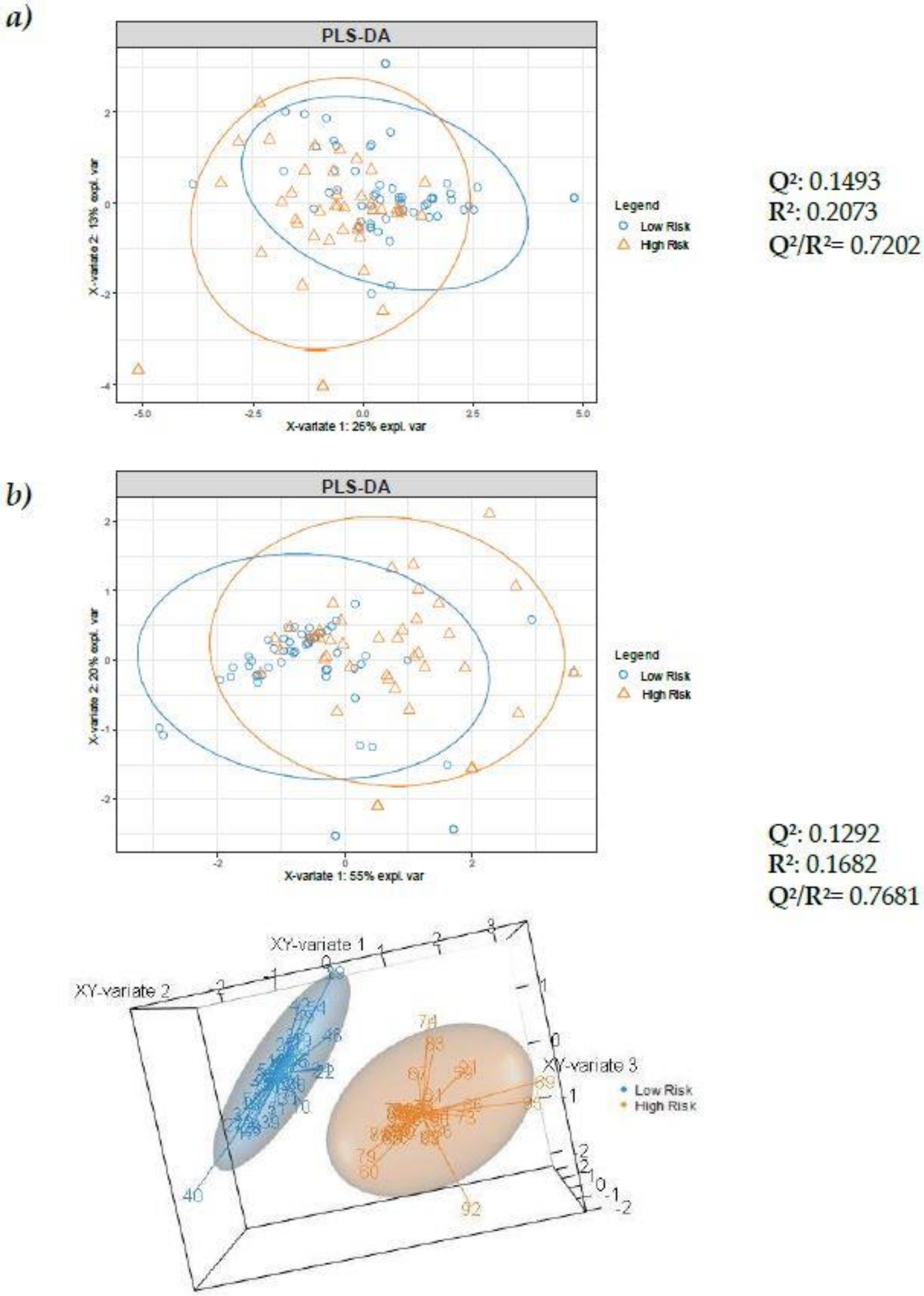

Figure S3. (**a**) Partial least squares discriminant analysis (PLS-DA) model in order to evaluate the potential of the 5 selected exo-oncomiRNAS plus PSA in serum, sTWEAK in semen, Age, Prostatic volume, Testosterone in the stratification of PCa aggressiveness. (**b**) PLS-DA model in order to evaluate the potential of the ROC model (sTWEAK in semen, exo-oncomiR-221-3p and exo-oncomiR-222-3p in semen) in 2-dimension and 3-dimension. Both models were evaluated using R2, Q2 and Q2/R2 metrics. A model is considered predictive when Q2/R2 ratio is greater than 0.5.

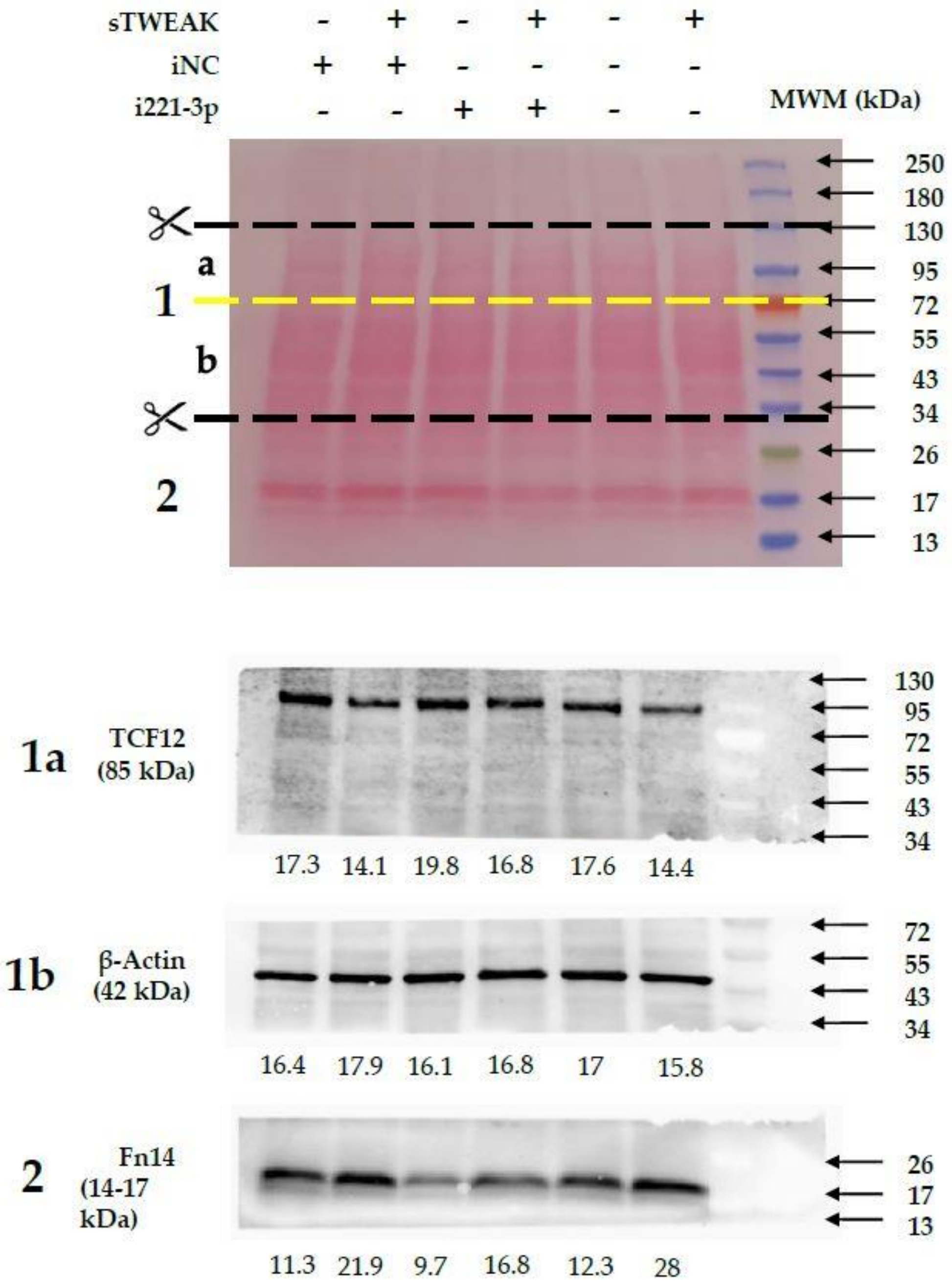
sTWEAK - + - + - +
iNC + + - - - -
i221-3p - - + + - -
MWM (kDa)
250
180
130
95
72
55
43
34
26
17
13
1
a
b
2
1a
TCF12
(85 kDa)
17.3 14.1 19.8 16.8 17.6 14.4
1b
β-Actin
(42 kDa)
16.4 17.9 16.1 16.8 17 15.8
2
Fn14
(14-17
kDa)
11.3 21.9 9.7 16.8 12.3 28

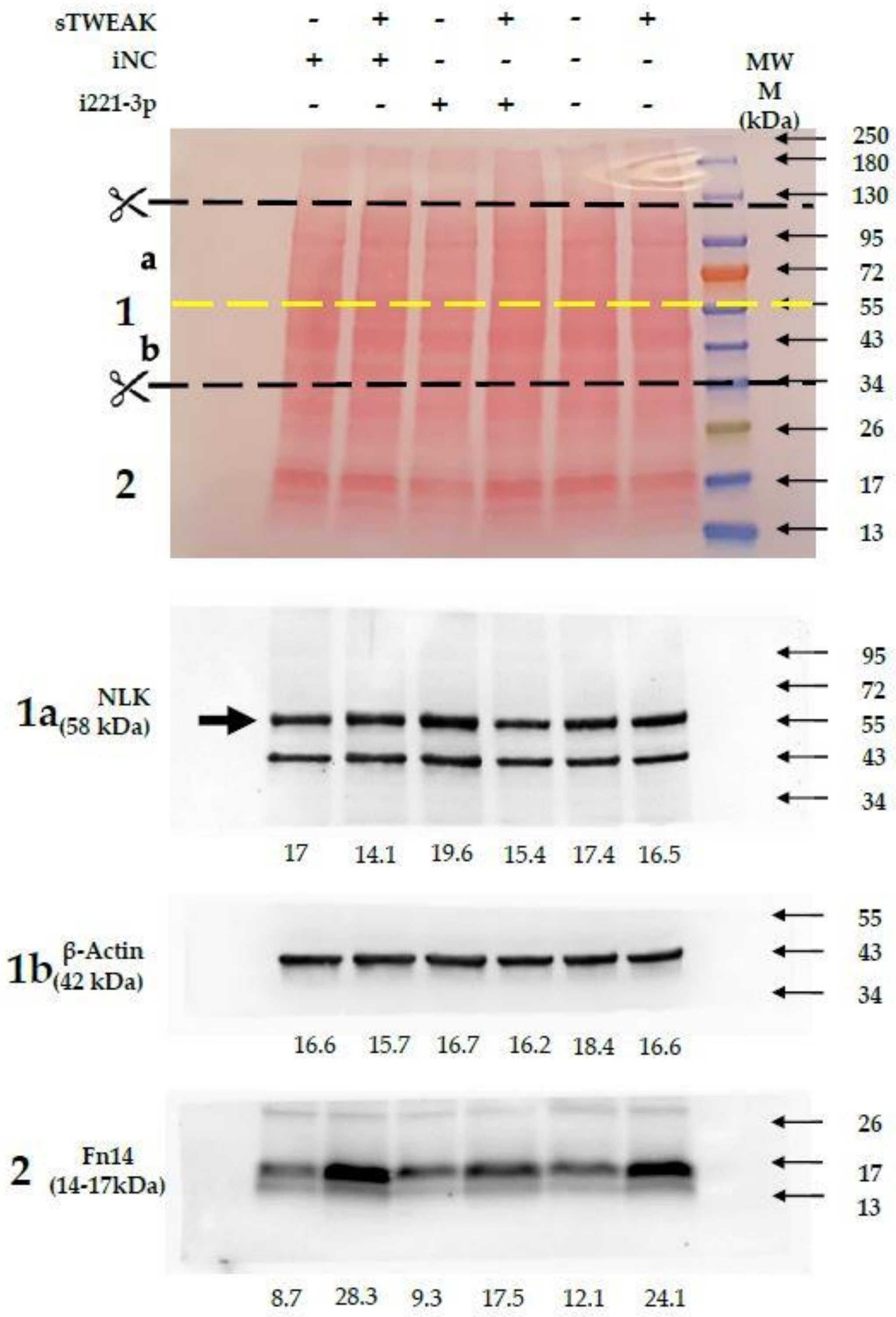

Figure S4. Complete WB results referring to Figure 4d. The membranes were cut before antibody incubation to allow multiple detection of a single gel without stripping off antibodies. Therefore, no complete membranes can be shown for this experiment. The numbers below membranes represent the percentage of intensity.

Publisher's Note: MDPI stays neutral with regard to jurisdictional claims in published maps and institutional affiliations

© 2020 by the authors. Licensee MDPI, Basel, Switzerland. This article is an open access article distributed under the terms and conditions of the Creative Commons Attribution (CC BY) license (http://creativecommons.org/licenses/by/4.0/).
